# An unusual case of a mature teratoma on the left perineal region of a young cat: surgical treatment and pathological description

**DOI:** 10.1186/1751-0147-55-51

**Published:** 2013-07-11

**Authors:** Ciprian Andrei Ober, Marian Taulescu, Liviu Oana, Lucia Bel, Cornel Cătoi, Laura Fărcas, Cosmin Pestean

**Affiliations:** 1 Department of Anaesthesiology and Surgery, University of Agricultural Sciences and Veterinary Medicine, Cluj-Napoca, Romania; 2 Department of Pathology, University of Agricultural Sciences and Veterinary Medicine, 3-5 Calea Manastur, Cluj-Napoca 400372, Romania

**Keywords:** Teratoma, Cat, Perineal region, Surgery, Histology, Pathology

## Abstract

A 10-month-old intact male cat with a clinical history of a large mass in the left perineal region was submitted to the surgery department. The mass had reportedly been present as a small swelling after birth. Cytological evaluation using a fine-needle aspirate showed eosinophilic keratinaceous debris, and was not convincing for the definitive diagnosis. Complete surgical excision was performed. Postoperative function and aesthetics were excellent. Based on gross and histological features the definitive diagnosis of the tumor was mature teratoma with ectodermal and endodermal components. After a follow-up period of 4 months, no signs of recurrence were evident. Surgical excision of the teratoma in our case was considered curative. A perineal location has not been previously reported in the cat and should be considered a rare condition in this species.

## Background

Teratomas are uncommon tumors containing elements that originate from more than one germ cell layer, are foreign to the organ in which they arise, and show independent growth [1]. Teratomas are characterised by the presence of tissue from more than one somatic cell type in contrast to dermoid cysts in which a single somatic cell type is identified [2]. Teratomas and germ cell tumors in general are mostly found in the gonads, but sometimes occur in the neck or along the midline of the brain, generally involving the suprasellar, thalamic, or pineal regions in both animals [3] and humans [4,5]. Few cases of feline teratomas have been reported in the literature [6-10]. The most feline teratomas are developed from the ovary or testicle because of their germ cell origin [6]. Extragonadal location of teratomas, such as intracranial [8], retrobulbar [10] and on the head [11] have also been reported in cats. The present paper describes the successful excision of a mature extragonadal teratoma located on the left perineal region of a kitten.

### Case presentation

**Figure 1:**
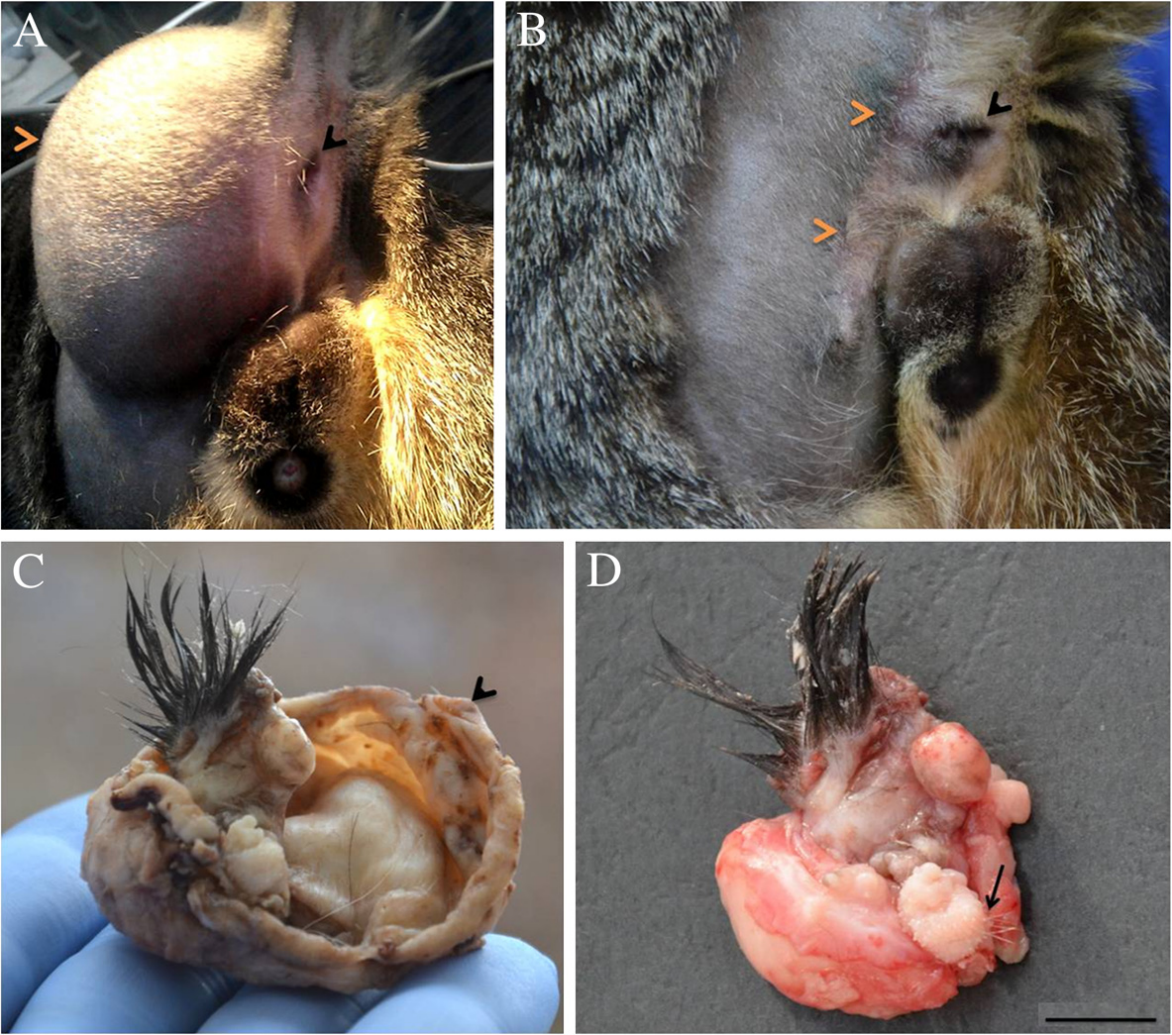
**Aspects of the perianeal area of the cat and gross aspects of the tumor. (A)** The cat at first presentation at 10 months of age. The tumor is located in the left perineal region (red arrow) and displaces the anus laterally to the right (black arrow); **(B)** Four months after surgery. Notice the good healing (red arrows) and the normal position of the anus (black arrow); **(C)** Photograph revealing the tumor mass which is surrounded by a white to gray fibrous capsule (arrow); **(D)** Photograph of the mass showing the multinodular aspects with two areas of hair arising from it (arrow).

A 10-month-old, domestic shorthair intact male cat was presented for examination with a clinical history of an approximately 3 cm in diameter soft and painless mass in the left perineal region (Figure 1A). A small swelling in this area had been present after birth, as reported by the cat’s owner. The mass continued to grow and caused intermittent constipation symptoms with mild tenesmus. Because of these, the owner came for diagnosis and treatment. No abnormalities were found during the rectal examination, and also both testicles were located in the scrotum. The X-ray showed a heterogenous structure and multiple foci of mineralization. No ultrasonography examination was performed at this time. Aspirates of the mass at various locations were stained using multiple Dia-Quick Panoptic stain (Reagent Ltd., Budapest, Hungary) and cytologically examined. These showed the presence of eosinophilic keratinaceous debris, not convincing for a definitive diagnosis. The decision was made to remove the tumor surgically. The cat was given xylazine (1 mg/kg intramuscularly) and induced with propofol (4 mg/kg intravenously (IV) to effect). An endotracheal tube was placed, and anesthesia was maintained with sevoflurane in oxygen, using a semi closed rebreathing system. Prior to surgery, the cat received clavulanate potentiated amoxicilline (10 mg/kg IV) and carprofen (3 mg/kg IV). During surgery, fentanyl 5 μg/kg per hour was administered. The mass was marginally removed according to the principles of oncological surgery [12]. A curvilinear skin incision was made over the bulging skin, 2 cm lateral to the anus, from the base of the tail to a point 2 cm under the pelvic floor. The tumor’s pseudocapsule (Figure 1C) was observed and the tumor was completely resected by blunt dissection. The mass was not adherent to any structures.

After lavaging the area, regional musculature was sutured with simple monofilament interrupted sutures (3–0 polydioxanone). The subcutaneous tissues were opposed with simple interrupted sutures (3–0 polydioxanone) and the skin was closed with simple interrupted no absorbable sutures (3–0 nylon) after a piece of skin was removed to eliminate the dead spaces and no drain was fixed.

The entire mass was submitted to the pathology department for gross and histological interpretation. The gross aspect was of a large cystic mass, red to gray, with round to oval shape, measuring 90×70×60 mm. On cut section, approximately 50 ml of yellow-red, fine granulated liquid mixed with several hair fragments leaked out and a multinodular, dense, red to gray mass, measuring 40×30×30 mm, was observed attached to inner surface of the cyst wall.

**Figure 2:**
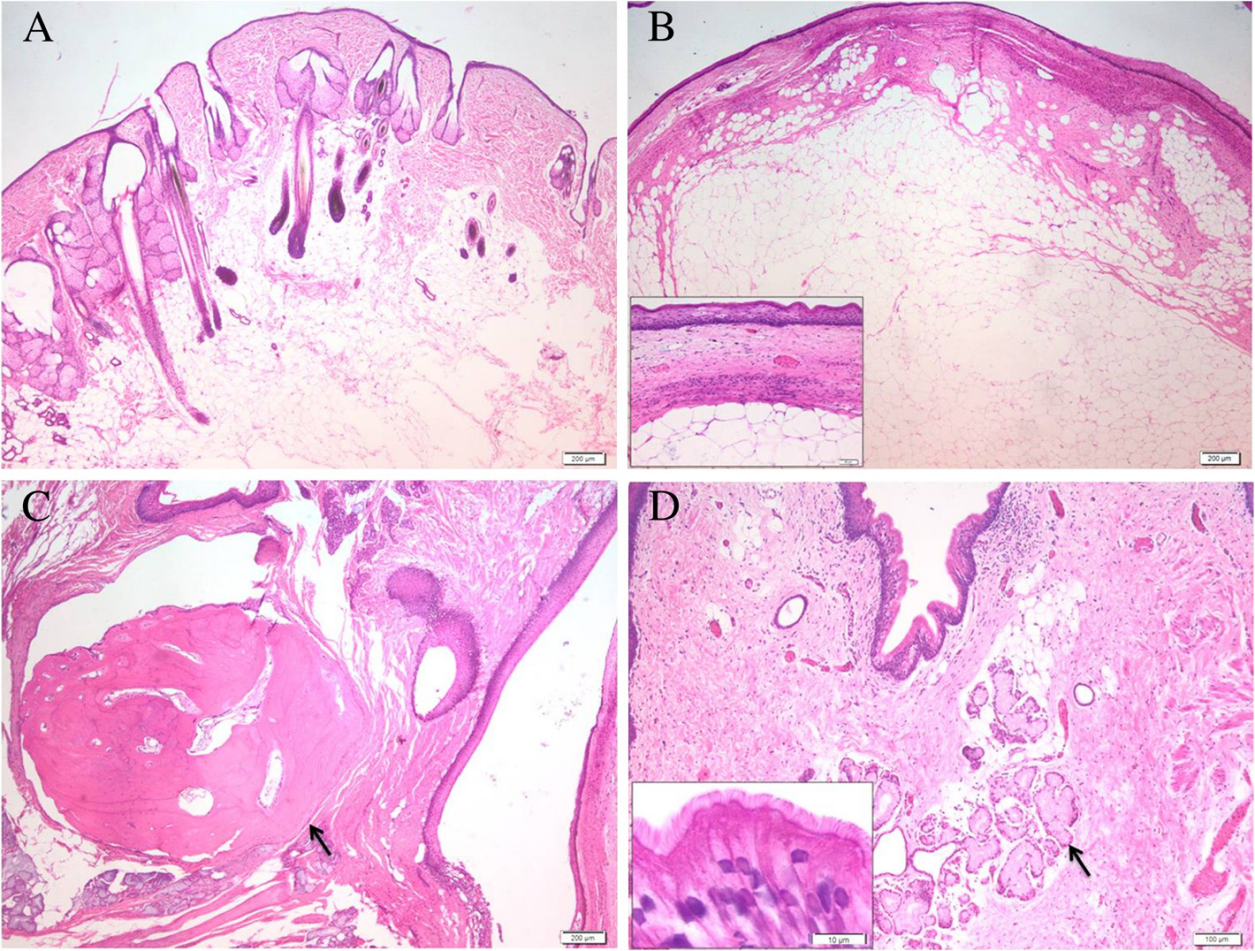
**Photomicrographs of haematoxylin and eosin-stained sections of the feline teratoma of a perianal region (HE stain). (A)** Normal skin with epidermal and dermal layers containing hair follicle, sebaceous, sweat gland units and adipose tissue (bar = 200 μm); **(B)** A nodular structure composed of adipose tissue surrounded by connective tissue and lined by squamous stratified epithelium (bar = 200 μm), the inset showing the detail of these structures (bar = 50 μm); **(C)** Island of woven bone resembling long bone (black arrow) (bar = 200 μm), and **(D)** Structures resembling respiratory tissue consisting of glandular components (black arrow) surrounded by connective tissue lined by columnar pseudostratified ciliated epithelium (bar = 100 μm), the inset showing the ciliated columnar epithelia (bar =10 μm).

An area with several long dark brown to black hairs was present on the surface of the inner mass. Another focal area with a few thin white hairs was also seen (Figure 1D). For histological examination, samples from both the cyst wall and inner mass were fixed in 10% phosphate buffered formalin for 24 hr, embedded in paraffin wax, cut into 3–5 μm sections, and stained with hematoxylin and eosin. The histological examination of the cyst wall showed a mature connective fibrous tissue without lining cells and was considered a pseudocyst. Microscopic analysis of the inner mass revealed a mixed structure consisting of skin and appendages (numerous hair follicles, hair bulbs, sebaceous and sweat glands) (Figure 2A), multifocal areas of adipose tissue (Figure 2B), islands of bone (Figure 2C) and hyaline cartilage, mature connective tissue, several glands lined by cuboidal to columnar epithelium with basophilic and foamy cytoplasm and pseudostratified ciliated epithelium (Figure 2D). No features of malignancy were seen in both cyst wall and inner mass. Based on gross and histological features the definitive diagnosis of the tumor was a cystic, mature teratoma with several components.

On the follow-up examination 21 days later, the wound had healed normally. No signs of local swelling, tenesmus or constipation were observed. After 4 months postoperatively the cat was presented for a routine examination, having a normal quality of life without any signs of recurrence.

## Discussion

Beside the fact that the incidence of the teratoma in cats is low (0.7% to 3.6%) [13], a perineal location has not been previously reported in this species. Also, to our knowledge in no other domestic species were perineal or retrorectal extragonadal teratomas reported.

The teratoma is based on primordial germ cells from the top cell layer of the blastocyst (the epiblast). From this arise the ectodermal, mesodermal and endodermal germ cell layers [14]. During embryogenesis, one of the primordial germ cells migrates from the yolk sac wall, over the primitive intestine and the mesentery to the roof of the coeloom. In this place they implant at the level of the so called paired genital ridges and form the gonads [15]. Extragonadal germ cell tumours can develop through the neoplastic transformation of cells that have not succeeded in reaching these genital ridges [10]. The neoplastic transformation of these cells is very rare [14]. In a normal situation extragonadal germ cells disappear in foeti older than 60 days [7]. In humans, sacrococcygeal teratoma is a neoplasm arising in the sacrococcygeal region [16] and there were suggestions that it originated from multipotential cells in Henson’s node, which migrates caudally to the coccyx. It is one of the commonest fetal neoplasms, but is rare in adults [17].

The content of teratomas is complex, reflecting their heterogeneity of germ-cell origin. Neural tissue, woven bone, hyaline cartilage, hair follicles, sebaceous and apocrine glands, respiratory epithelium and adipose tissue have all been reported in animals [8,9]. Histologically, three embryonic components with different tissues were identified. In contrast to other reports [10,11] which have identified ectodermal and mesodermal components as predominant structures of head and retrobulbar teratomas, in our case ectodermal and endodermal components were mostly identified.

Based on the degree of malignancy, the tumor had a benign nature pathologically and clinically, and the cat recovered completely after surgery. Teratomas with immature (poorly differentiated) histological components are often referred to as teratocarcinoma [13].

Norris (1969) describes a solid teratoma in a cat with dysgerminomas; the tumor grew invasively and extended through the abdominal wall [18]. Although malignancy and metastasis have both been noticed in cats [8], in our case no malignancy signs were identified.

According to other clinical case reports [10,11,15] cytological examination was not sufficient to diagnose the type of the tumor because of contamination of the sample with non-diagnostic fluid from the cyst. In our case only eosinophilic keratinaceous debris were found and the sample was considered to be nondiagnostic.

Although, the biopsy is recommended before surgical tumor excision for definitive diagnosis [12], the owner wasn’t agree with the procedure. The X-ray was the only paraclinical exam used for a presumptive diagnosis of a heterogeneous structure with mineralization of the perineal mass.

A differential diagnoses should be considered from perineal hernias in cats. Although a rare condition in cats, mostly in neutered males and typically bilateral [19], some cases of perineal hernia have been documented [20-22]. It is very difficult to establish the definitive diagnosis based only on clinical information. Thus, history, digital rectal examination, radiology, ultrasonography, centesis (for bladder retroflection), cytology and biopsy are required to differentiate these two conditions.

In humans, primary retrorectal teratomas are also very rare and in addition to the anatomical position of tumors, difficulty in diagnosis and surgical management often occur [23]. Even though some tumors are malignant [24], most are benign, leading in some cases to chronic constipation [25]. Most of the retrorectal tumors will also require surgical resection.

Because of intermittent constipation with tenesmus it was suggested that surgical excision provided a chance for local tumor control and clinical signs improvement. Four months after surgery the good healing of the tumor site, the normal position of the anus, good clinical condition and abdominal ultrasonography indicated no metastases in our case (Figure 1B). This mature (benign) teratoma caused clinical signs only because of its size and perineal location. The prognosis of the tumor was good because by blunt dissection close to the tumor pseudocapsule, the well delimited mass was entirely removed with the preservation of essential anatomical elements and the overlying skin. We agree with a recent report [11] that the young age at presentation in these cases is most likely due to the extragonadal location, where tumors are more easily recognized by the owner. Although, in our case, the owner observed a swelling in the left perineal region after birth, he delayed presentation at the veterinarian, until he saw the tumor starting to grow and entail clinical problems.

## Conclusions

Although this is a rare tumour, it should be included in the differential diagnosis for a perineal swelling in a young cat. Complete surgical excision has the potential to be curative in case of a mature teratoma.

## Competing interests

The authors declare that they have no competing interests.

## Authors’ contributions

CAO and CP performed the anesthesia and surgery; although CAO is the main author of the paper. MT and LF performed the histopathologic examination and interpretation. LB is the referring clinician for the present case. LO and CC reviewed the paper. All authors read and approved the final manuscript.
